# Wearing Time of Ankle-Foot Orthoses with Modular Shank Supply in Cerebral Palsy: A Descriptive Analysis in a Clinically Prospective Approach

**DOI:** 10.1155/2019/2978265

**Published:** 2019-07-15

**Authors:** M. Schwarze, L. Horoba, J. Block, C. Putz, M. Alimusaj, S. I. Wolf, T. Dreher

**Affiliations:** ^1^Clinic for Orthopedics and Trauma Surgery, Center for Orthopedics, Trauma Surgery and Spinal Cord Injury, Heidelberg University Hospital, Germany; ^2^Pediatric Orthopaedics and Traumatology, University Children's Hospital Zurich, Zurich, Switzerland

## Abstract

**Objective:**

To date there is only limited knowledge about the wearing time of orthoses. Ankle-foot orthoses (AFOs) have not been studied with this research question. Additional influences of the orthotic design as well as weekdays and the weekend are also unknown.

**Design:**

Monocentric, clinically prospective intervention study.

**Patients:**

Inclusion of 10 patients with bilateral spastic cerebral palsy.

**Methods:**

Equipment of all subjects with a dynamic ankle-foot orthosis (DAFO) and modular shank supply (MSS, dynamic elastic shank adaptation or ground reaction AFO). Integration of temperature sensors for recording the wearing time for a period of 3 months.

**Results:**

The actual wearing time was below the recommendations on actually worn days as well as the average of the entire study period. In addition, the actual usage in terms of days and hours was well below the recommendations. The wearing time showed differences between weekdays and weekend. Differences between DAFO and MSS were not detectable.

**Conclusion:**

The actual usage behavior of ankle-foot orthoses differs from the recommendations of the prescriber. This applies to both DAFOs and modular use with shank supplies. Environmental factors may have a significant impact on wearing times on weekdays and the weekend.

## 1. Introduction

With a prevalence of 1.4-2.3‰, cerebral palsy is the leading cause of motor impairment in children in western civilization [1] Orthoses, especially ankle-foot orthoses (AFOs), play an important role as part of conservative therapy concepts in all age groups. Their importance, however, is underlined in particular in the collective up to the age of about 6 years due to the dynamic nature of the condition [2]. These pursue the goal of individual support for compensating associated impairments in postural and selective motor control as well as concomitant spasticity and muscular weakness to enable culturally appropriate activities and participation. Support of the foot as a base is necessary due to a prevalence of more than 90% of foot deformities and aberrant gait patterns in children with cerebral palsy [3]. In a French cross-sectional study Sacaze et al. described that up to 85% of patients use at least one assistive device, with nighttime ankle-foot orthoses being the most common at 43.6% in their collective [4].

In the scientific debate on advantages and disadvantages of orthoses of the lower extremity, at the present time the main focus is on biomechanical influences on the individual gait pattern. Based on decades of research, numerous positive kinematic and kinetic effects have been described. These include, for example, an increase in walking speed and stride length, decrease in cadence, and reduction of oxygen consumption. Positive influences on the ankle, knee, and hip are known especially in the sagittal plane. Conclusively, positive changes in the gait indices Gillette Gait Index, Gait Profile Score, and Gait Deviation Index were described [5–8]. Additional positive effects after multilevel surgery have been reported recently [9]. Unfortunately, according to the last ISPO Cerebral Palsy Consensus Conference, the study results are partly contradictory and lacking long-term studies [10, 11].

Undoubtedly, however, it must be stated that, in order to achieve any effects, the orthoses have to be worn. Despite all the discussion about possible effects, there has been no relevant focus on the actual use of these orthoses. This is unfortunate, since it is the desire to improve the level of activity that drives patients to their practitioners [12]. To the best of our knowledge, to date there are only three studies investigating wearing time of lower extremity orthoses in the context of underlying neurological diseases [13–15]. This is insufficient, since it can be assumed that the clinical benefit is also significantly dependent on the wearing time [16]. In clinical health services research, the investigation of wearing time of assistive devices began just a few years ago. On the basis of different measurement methods such as questionnaires [17], force transducers [18, 19], and temperature sensors [20, 21], which are regarded as gold standard [18], corsets in scoliosis were the first examined assistive devices in clinical focus. Study results revealed impressively that on the one hand the compliance regarding wearing time ranged only between 27% and 47%, although, on the other hand, a clear negative correlation between the time in brace and the likelihood of surgery could be demonstrated [17, 22, 23]. In one of the few transductions of this methodology to lower extremity orthotics, Maas et al. described relevant differences between sensor based and parental information regarding wearing time, in a study of knee-ankle-foot orthoses (KAFOs) in children with cerebral palsy [14]. In clinical practice, however, at 0.7%, these account for only a very small proportion of all assistive devices [4]. The aim of this study was therefore to investigate the wearing time of the more commonly used ankle-foot orthoses in children with cerebral palsy. Since it can hypothetically be assumed that the therapy adherence is also influenced by the design of the orthosis (especially modularity), this factor has also been brought into the focus of the study. Furthermore, it is of interest whether wearing time differs between weekdays and the weekend since it can be assumed that the level of activity, individual requirements, and also peer influences differ.

## 2. Material and Methods

The study design was monocentric, nonblinded, clinically prospective. Ethical approval was given by the ethics committee of the Heidelberg University's Faculty of Medicine on November 11th in 2015 (S-460/2015).

### 2.1. Subjects

Recruitment took place in the neuroorthopedic department of our university hospital. We included patients with bilateral spastic cerebral palsy (BSCP) in an age range of 4 to 10 years at baseline. Another inclusion criterion was a GMFCS level [24] of 1-3. Patients with structural knee flexion contracture were excluded. There were no further restrictions concerning underlying foot deformities or gait patterns. All criteria were checked during an outpatient clinical examination. An extensive medical examination by an experiences pediatric orthopedist resulted in a reasoned medical indication for a modular orthosis as combination of a dynamic ankle-foot orthosis (DAFO) and modular shank supply (MSS). Indications for this combination were the underlying foot deformities and the respective CP-typical gait pattern. Additionally, calf muscle weakness and level of spasticity were taken into account for being able to control foot position in the sagittal plane, leading to an adequate control for proximal joints while standing and walking.

All participants and their legal guardians were fully informed about the study and gave written informed consent about participation. In total 10 patients met the inclusion criteria during the recruitment period between April 2016 and June 2017.

### 2.2. Orthotics

**Figure 1:**
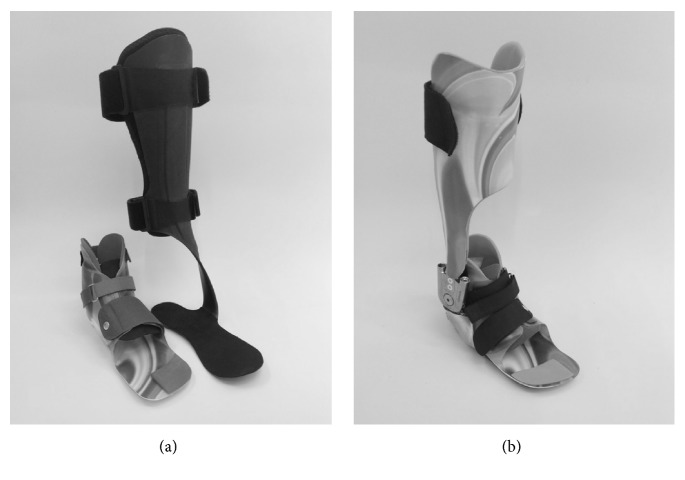
(a) DAFO with ToeOFF adaptation; (b) GRAFO including DAFO.

The following orthotic designs were used:

1. DAFO (Figures 1(a) and 1(b)), known as Nancy Hylton orthosis [9, 25]: orthosis made of thin, flexible thermoplastic polypropylene surrounding the foot up to the ankle joint. Despite a lack of scientific evidence of the latter mentioned effect, the special, custom-made footplate supports the dynamic arches of the foot and is intended to introduce proprioceptive stimuli on a reflex-based level to achieve a detonating effect [26, 27]. Furthermore, it has a corrective static effect on the foot deformity and the instability.
2. Indication: underlying combination of mechanical and/or motor control deficits which result in dynamic and/or structural deformities that lead to functional foot instability, e.g., lever arm dysfunction.

All study participants were equipped with a DAFO. Depending on the individual needs (gait disorder, foot deformity, height, and body mass), one of the following modular shank supplies (MSS) was additionally used in a modular orthotic design (DAFO + DESA or DAFO + GRAFO).

1. Dynamic elastic shank adaptation (DESA, Figure 1(a)) [9]: orthosis with anterior tibial attachment (ToeOff, Allard USA Inc., USA). This off-the-shelf orthosis consists of a thin carbon fibre foot plate, which is inserted into the footwear and is connected via a spiral lateral-side carbon fibre composite lamella with the anterior shin system. Deformation of the material generates resilient restoring forces. A beneficial effect on the plantar flexion-knee extension couple is expected. The adaptation was aligned by sole wedges with respect to the manufacturer's recommendations.
2. Indication: calf muscle weakness and/or impaired muscle control resulting in reduced knee stability in the sagittal plane, e.g., crouch gait or stiff gait resulting in the need of mild to moderate support and fore foot lever arm restoration. Additionally, need for support of the ankle positioning towards neutral position, if DAFO alone is not sufficient.
3. Ground reaction force AFO (GRAFO, Figure 1(b)) [9]: custom-made carbon orthosis with semicircular anterior tibial shell and unilateral hinge joint at the ankle. Adjustable dorsiplantar stops are used to limit the range of motion (hinged AFO [HAFO]) and combined with springs for a slight support of toe lift [28]. The primary effect is expected at the dorsal stop by producing a defined ankle moment and, indirectly, by a knee-extending moment via the plantar flexion-knee extension couple, as described previously [29]. A higher stabilization in all three planes is achieved by a rather rigid structure. In particular the foot lever is stabilized in such orthoses, via circular containment of the foot.
4. Indication: calf muscle weakness and/or impaired muscle control resulting in reduced knee stability in the sagittal plane, e.g., crouch gait or stiff gait resulting in the need of moderate to high support and fore foot lever arm restoration. Additionally, need of increased support of ankle positioning towards neutral position, if DAFO + DESA are not sufficient. Hinge joints enable the options of increased control of range of motion and ankle positioning by adjustable dorsiplantar stops and allow support of dorsiflexion in swing phase by integrated springs [28].

Figure 1 (Figure 1) illustrates all orthotic designs.

With the exception of DESA, orthoses were built according to standardized protocols by experienced, certified orthotists in the technical orthopedics department and subject to individual medical control of fit and adjustment. All study participants and their parents were informed that they have the option of follow-up visits due to any kind of problems with the orthoses at any time.

### 2.3. Measurement of Wearing Time

Temperature sensors (orthotimer®, rollerwerk medical engineering & consulting, Balingen, Germany) were placed in straps or pads of the orthosis as closely as possible to the skin surface. Due to the modular design of all included orthoses, the independently usable modules (DAFO + MSS) were separately equipped with a sensor. Due to the bilateral affection and orthotic equipment of all patients, the orthosis components from only one randomly selected side were equipped with sensors.

The sensors measure the local temperature every 15 minutes for 3 months and store data on an integrated memory. Based on the manufacturer's recommendations, periods in which the measured values ranged between 29 and 38.5°C were rated as “wearing time”. The sensors could be read out wirelessly (certified according to ISO 15693) and were removed from the orthoses after the end of the study. Microsensor, reader, and software are CE class 1 (MDD2007/47/CE) and FDA approved.

All subjects were informed about the presence of the temperature sensors.

### 2.4. Study Protocol

At T1, all 10 patients received their new modular orthotic device which was checked for function and fit. Based on the recommendation by Tardieu et al. [16], they were instructed to use the orthosis for at least 6 h per day during their activities of daily living for a period of 3 months.

An integral part of the study was the instruction to the participants to use the modular prosthetic design in a weekly change. They should use DAFO alone for one week and the DAFO + MSS for the following week. In this way a uniform adaptation to both designs should be ensured.

Due to technical issues 6/10 sensors from the DAFOs and 8/10 sensors from the shank adaptation generated a complete data set at T2 and were included in the evaluation.

### 2.5. Statistical Analysis

The statistical evaluation was based on advice from the recommendations of the Department of Medical Biometry of the university hospital.

SPSS version 24 (IBM Germany GmbH, Ehringen, Germany) was used for the statistical analysis. Due to the explorative nature of the study, the small number of cases, and additional drop outs, a purely descriptive data analysis was performed, specifying mean (M), standard deviation (SD), minimum (min), and maximum (max).

Based on the daily wearing time and weekly change recommendations, the recommended wearing time was calculated knowing the average time between measurements T1 and T2 (108 [SD 20] days).

## 3. Results

### 3.1. Patient Population

The study included 10 children (3 ♀, 7 ♂) with an average age of 7 (SD 3) years. At T0, the average body height was 115.8 (SD 17.7) cm with an average body mass of 21.34 (SD 9.76) kg. At T1, they averaged 117.8 (SD 18.3) cm at 21.79 (SD 10.55) kg.  Table 1 (Table 1) illustrates the individual type of MSS, GMFCS levels, underlying foot deformities as well as the gait pathologies according to the Rodda classification [30].

### 3.2. Wearing Time

**Figure 2:**
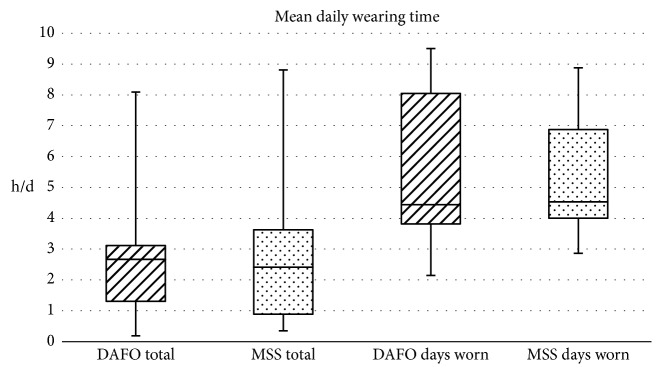
Mean daily wearing time for DAFOs and MSS in total and for the days worn.

The data analysis revealed heterogeneous wearing times in between the DAFO and MSS users. Only one out of six DAFO and one out of eight MSS participants wore the orthosis according to the recommended daily wearing time, but not a single patient wore the orthosis on every single day during the measurement period.  Figure 2 illustrates the mean daily wearing time for DAFOs and MSS in total and separately for the days the orthoses have been worn in the form of boxplots.

**Figure 3:**
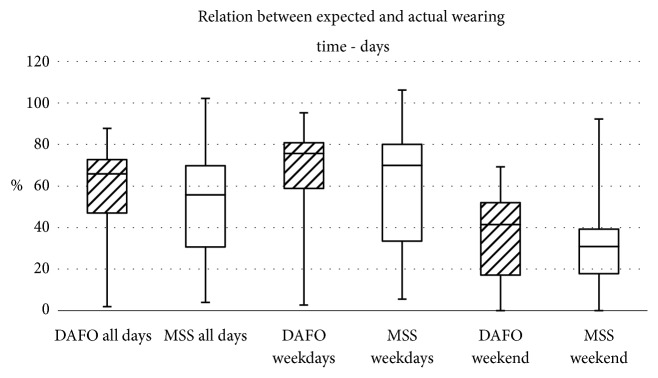
Relation between expected and actual wearing time with focus on the days worn.

**Figure 4:**
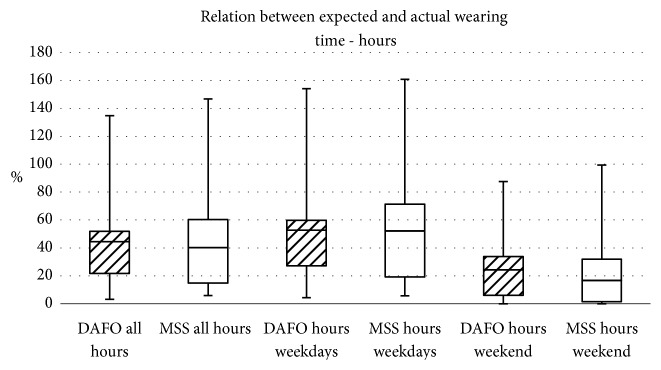
Relation between expected and actual wearing time with focus on the hours worn.

Figures 3 and 4 show the relation between expected and actual wearing time for days and hours.

**Figure 5:**
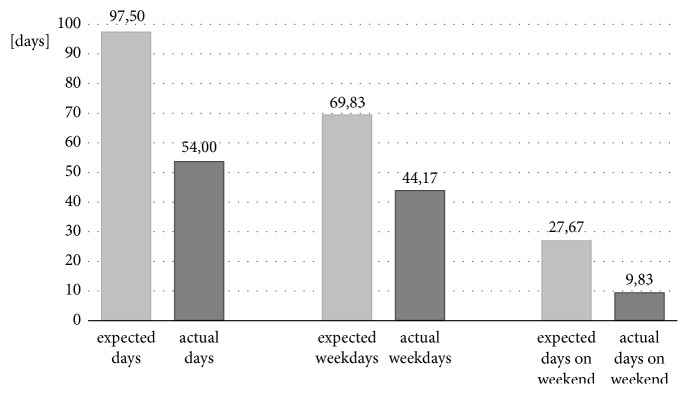
Descriptive analysis of expected and actual days worn in relation to all days, weekdays, and the weekends for DAFOs.

**Figure 6:**
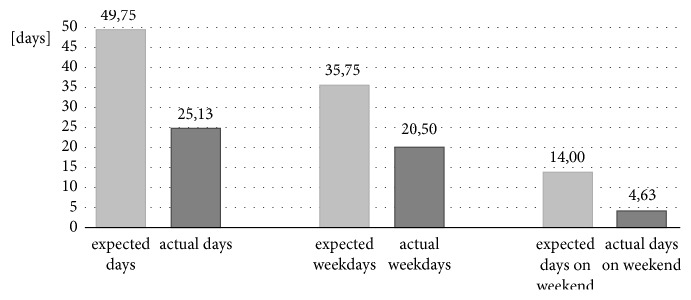
Descriptive analysis of expected and actual days worn in relation to all days, weekdays, and the weekends for MSS.

Figures 5 and 6 illustrate the difference between the expected days of use and the days when the orthoses were actually worn for the DAFOs and the MSS.

**Figure 7:**
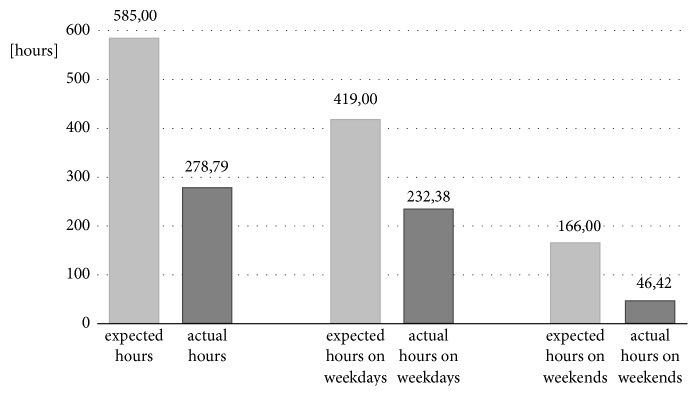
Descriptive analysis of expected and actual hours worn in relation to all days, weekdays, and the weekends for DAFOs.

**Figure 8:**
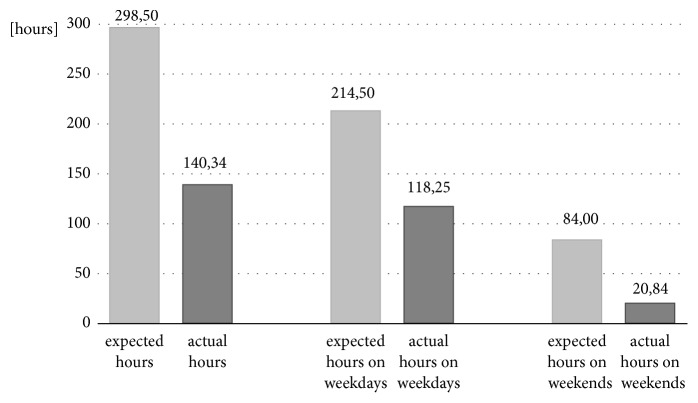
Descriptive analysis of expected and actual hours worn in relation to all days, weekdays, and the weekends for MSS.

Figures 7 and 8 show the difference between the expected hours of use and the hours when the orthoses were actually worn for the DAFOs and the MSS.

The data indicated a different wearing behavior between weekdays and weekends and a somewhat lower compliance in the use of MSS. The direct comparison between the two modular orthotic components revealed no pronounced differences in wearing behavior.

## 4. Discussion

The descriptive evaluation of the wearing time of DAFOs and MSS implies that there is a considerable discrepancy between expected and objectively measured use of the orthoses. In addition to the very heterogeneous and interindividually very fluctuating average daily wearing time, context factors also seem to have a relevant influence on it. This can be deduced from the very different usage behavior between weekday and weekend. The impact of the modular orthotic design seems to be negligible.

Detailed knowledge about therapy adherence is a prerequisite in the discussion of possible therapeutic effects of ankle-foot orthoses. At the present time, however, there is a relevant lack of scientific data at this point so that every prescriber is faced with the uncertainty of not knowing how often and how long prescribed orthoses are actually worn. The data collected as part of our three-month prospective study protocol illustrates that there are relevant differences even in a small but very homogenous group of patients.

On the one hand, corrective and stabilizing and, on the other hand, preventive purposes play a significant role in the basic therapeutic thoughts on AFOs [9]. In 1988, Tardieu et al. published data from a study that sought to find out how long the soleus muscle should be stretched daily to avoid contractures. The authors came to the conclusion that daily stretching of at least 6 hours would avoid contractures, whereas stretching as short as 2 hours could cause progressive contractures [16]. The recommendations derived from this publication are still frequently recited in current scientific literature when recommending daily orthotic wearing time. Although ultimately the transferability of this recommendation to other muscle groups or different medical conditions is unclear, there is to date no generally accepted differing therapy recommendation.

In the survey of wearing time of assistive devices, the selected methodology is a significant influencing factor. In 2018 Maas et al. described a bidirectional misinformation in the parental interview in a study of the wearing time of knee-ankle-foot orthoses [14]. Compared to objective measurements via temperature sensors, they over- or underestimated the real wearing time. However, the former statement relativizes ex post the data published by the same research group in 2014 [13]. At that time, they recorded wearing times of KAFOs as nighttime splints as secondary outcome parameter based on questionnaires and temperature sensors in 12 participants with spastic cerebral palsy in a randomized controlled trial [13]. The results showed an average wearing time of 3 h (SD 0.9h) per night. However, as only 5 of the 12 measurements were taken with temperature sensors and the rest was based on questionnaires, the results are somewhat biased. A similar discrepancy between self-reported and objective measurement has also been observed in other fields of treatment, such as clubfoot and scoliosis therapy [17, 31].

Although the results presented by Maas et al. seem quite comparable to ours, the comparison is fundamentally limited. The authors used KAFOs as nighttime splints, which represents a significant difference in indication to our AFOs. These are worn as part of the daily activity to directly support the patients during their activities of daily living. However, due to the lack of comparable studies that measured the wearing time of orthoses objectively, a different comparison is not possible.

In 2013, Zhao et al. published data on the wearing time of AFOs [15]. In a prospective randomized controlled trial, the authors included 112 ambulatory children with spastic diplegia and compared effects of day vs. day-night use. The wearing time was recorded by questionnaires. In summary, they came to the conclusion that day use of AFOs was more effective in improving Gross Motor Function Measure scores than the day-night use and that prolonged wearing may influence muscle activity. Their evaluation of wearing time resulted in 6.8h per day in the day group and 19.4h in the day-night group. Their day group thus showed slightly higher values compared to the measurement data of our cohorts on the days when the orthoses were actually worn.

The examination of the results of our investigation indicates further interesting characteristics in the usage behavior of AFOs. Apart from the known fact that the actual wearing time deviates from the recommendations, there seems to be a relevant difference between weekdays and the weekend. In the descriptive presentation, there was a big difference in both the percentage daily and the percental hourly usage. This was observed in both DAFOs and MSS. To the best of our knowledge, such differences have not described in the literature before. We hypothesize that the change from weekday to weekend routines has a direct impact on wearing time due to different motor requirements. Possibly, on weekends activities with lower motor demands, such as playing on the ground, could prevail over weekday activities such as school sports. This might make wearing an orthosis in the individual consideration of any advantages and disadvantages dispensable. This effect seems to exist for both DAFOs and MSS. The modular orthotic design chosen in our study usually pursues the goal of dynamically adapting the orthotic function to the user's individual demands and needs. However, this adaptive additive support seems dispensable at the level of the ankle and lower leg. Relevant differences in wearing behavior were not observed.

## 5. Limitations

The overall message of this study is limited by the small number of study participants. Strict, well-defined inclusion criteria should ensure a very homogenous group of subjects with bilateral spastic cerebral palsy. However, this was to the detriment of the number of cases.

The recommendation of a weekly change in the orthotic care does not correspond to the usual clinical approaches. This should ensure, with regard to possible biomechanical evaluations, that a uniform habituation to both supply strategies was guaranteed.

Technical issues with the used temperature sensors further limit the results due to 4 drop outs in the AFO (40%) and 2 drop outs in the MSS group (20%) because of incomplete or unavailable data sets. Since only complete data records should be included in the evaluation, the decision was made to keep the analysis purely descriptive. However, this in turn considerably limits the possible conclusions to be drawn from the illustrated results. All observations are purely descriptive and require further investigation with larger number of cases and more comprehensive statistical analysis. In addition, an examination of the relationship between wearing time and subjective or objective benefit would be helpful.

The unblinded design of the study included the use of temperature sensors in the wearing time survey. All participants were fully informed about this procedure. Subjects adapt their behavior when knowing that they are being studied. This is described by the so-called Hawthorne effect [32]. In the context of this effect, however, it is all the more remarkable to see the variations in wearing times.

Even if the use of temperature sensors is considered most feasible in the survey of wearing time, no level of activity can be derived from the elevation of the temperature [18]. It is also conceivable that the temperature range of the sensors, which was interpreted as “wearing time”, was not reached by using the orthoses in the case of corresponding climatic conditions. To avoid such misinterpretations, additional motion sensors would be required.

## 6. Conclusion

The mean daily wearing time of DAFOs and MSS is below the recommendations and shows remarkable heterogeneity. Relevant differences can be observed comparing weekday and weekend for both designs. DAFO and MSS showed the same tendency in wearing behavior without relevant difference in the mean daily wearing time.

## Figures and Tables

**Table 1:** Patients' characteristics including MSS, GMFCS level, foot deformities, and gait pattern according to the Rodda classification [30].

Patient	MSS	GMFCS	Gait pattern	Foot deformity right	Foot deformity left
1	DESA	3	Crouch gait	Pes planovalgus	Pes planovalgus

2	GRAFO	2	True equinus	Pes equinus et planus	Pes equinus et varus

3	DESA	2	Crouch gait	Pes planovalgus	Pes planovalgus

4	DESA	2	Crouch gait	Pes planovalgus	Pes planovalgus

5	DESA	1	Crouch gait	Pes transversoplanus et varus	Pes planus

6	DESA	1	Jump knee	Pes planovalgus et transversoplanus	Pes planovalgus, transversoplanus et adductus

7	DESA	2	Jump knee	Pes planovalgus	Pes planovalgus

8	DESA	2	Crouch gait	Pes planovalgus	Pes planovalgus

9	DESA	2	Crouch gait	Pes planus et transversoplanus	Pes planus, transversoplanus, varus et adductus

10	DESA	2	Crouch gait	Pes planovalgus	Pes planovalgus

## Data Availability

The data in this study are not permanently freely available online. Upon request, however, they can be made available after contacting the corresponding author at any time.
